# Supplementary material for: SyNDI: synchronous network data integration framework
Source: BMC Bioinformatics. 2018 Nov 6;19:403. doi: 10.1186/s12859-018-2426-5 (PMC6219086; doi:10.1186/s12859-018-2426-5)
Supplement: Supplementary file 5 — Files of the example presented in Additional file 4. This additional file is a zip package that contains all files used in Additional file 4. Detailed descriptions of each of these files are presented in a README file included in this zip package. (GZ 30411 kb) [file 12859_2018_2426_MOESM5_ESM.gz › SyNDITools/Meme2Fimo/meme_4.9.0_4.tar.gz!/meme_4.9.0/doc/glam2_alphabet.html]

GLAM2 Alphabets


# GLAM2 Alphabets

## Alphabet files

Alphabet files allow glam2 and glam2scan to operate on sequences
over arbitrary, user-defined alphabets. They also allow residue
abundances to be specified. Their format is inspired by that of vmatch. For examples, see
robinson.alph and dna.alph in the GLAM2 examples directory.

**Note:** as soon as you specify
an alphabet file, the glam2 programs lose all knowledge about residues'
tendencies to align with each other. So, if you use an alphabet file to
specify amino-acid or nucleotide abundances, you probably want to
specify a Dirichlet mixture file too:
recode3.20comp or glam\_tfbs.1comp.

In alphabet files, the # character introduces a comment:
everything from it to the end of the line is ignored. Otherwise, each
non-blank line defines a symbol of the alphabet. The first
non-whitespace character on the line is the main character representing
the symbol: this is how the symbol is printed. Any characters that
follow it without any whitespace are aliases (when reading input). This
is optionally followed by whitespace and then a number, indicating the
abundance of the symbol. The abundances can be counts, fractions, or
percentages: they will be normalized so that they sum to 1. Unspecified
abundances default to 1. The final symbol is the *wildcard*: it
is forbidden from appearing in aligned columns, and all characters not
defined in the alphabet file are aliases of it. No abundance is defined
for the wildcard (any number will be ignored).

The order of the symbols matters when reading Dirichlet mixture
files or looking at reverse strands.

## Built-in alphabets

The p (protein) alphabet is equivalent to using robinson.alph
(and recode3.20comp) in the GLAM2 examples directory. The n (nucleotide)
alphabet is equivalent to using dna.alph (and glam\_tfbs.1comp) in the
GLAM2 examples directory.

## FASTA format

When reading sequences in FASTA format, the > character begins
the title of the next sequence, which continues till the end of the
line. In the sequence itself, whitespace is always ignored, and
non-whitespace characters are always part of the sequence: if not
defined in the alphabet file, they are interpreted as wildcards.

## Reverse strands

glam2 and glam2scan provide options to look at both strands of
the input sequences. This may only be meaningful for nucleotide
sequences, but is actually defined for all alphabets. The reverse strand
is obtained by first reversing the sequence, and then swapping each
symbol with its opposite in the alphabet's order (apart from wildcards).
Thus, for nucleotides, these symbols are swapped: a:t and c:g. For
proteins, these symbols are swapped: A:Y, C:W, D:V, E:T, F:S, G:R, H:Q,
I:P, K:N, and L:M.
